# Action game experimental evidence for effects on aggression and visuospatial cognition: similarities, differences, and one rather foolish question

**DOI:** 10.3389/fpsyg.2014.00088

**Published:** 2014-02-07

**Authors:** Christopher J. Ferguson

**Affiliations:** Department of Psychology, Stetson UniversityDeLand, FL, USA

**Keywords:** video games, aggression, violence, cognition, children and adolescents

## Introduction

We are beginning to understand that the social sciences often leap beyond the data, ignore null effects and overstate confidence in cherished beliefs (Ioannidis, 2005; Pashler and Harris, 2012). When perceived health of children is involved, this general effect can be exacerbated into *crusade bias;* the tendency to distort, overstate, or misrepresent research findings to lend a veneer of science to a polemic social agenda. That this occurred in the field of media violence has been well established (Savage, 2004; Sherry, 2007; Ferguson, 2013). But, with media, a parallel process which we might call a *savior bias* also emerges in which the media are considered a remarkable game-changer for reinventing society (e.g., McGonigal, 2011).

In a few short years, research on action games and aggression has gone from an “absolute truth” (e.g., American Psychological Association, 2005) to a full-blown replication crisis. In this essay I examine the degree to which the field of action games and visuospatial cognition may run similar risks. I wish to be clear that, in the debate on visuospatial cognition research, I respect researchers on both sides, and I hope that my comments may be viewed as constructive suggestions for improving the field, rather than merely as criticisms. With that in mind, here are several observations.

## Similarities between aggression and visuospatial research

### External validity

Both fields rely heavily on outcome instruments that do not transfer well to the real world. The aggression literature has been seriously plagued by this issue for some time (Savage, 2004; Elson, 2011). Regarding visuospatial cognition, many studies examine the influence of action games on interesting but esoteric laboratory tasks of visual attention and processing (e.g., Green and Bavelier, 2006; Blacker and Curby, 2013). It is not clear that the field has made the next step into demonstrating practical value of these laboratory effects. My concern has been exacerbated by difficulty replicating these findings myself using what I considered measures of visuospatial intelligence closer to what parents or policymakers might be interested in (e.g., Valadez and Ferguson, 2012; Ferguson et al., 2013). In fairness, some research has indicated that surgeons who play action games are better at certain types of surgery (e.g., Rosser et al., 2007). Yet it is not clear that how this research can be generalized to outcomes of practical value has been well-delineated.

### Adequate control conditions

Many video game studies of aggression introduced systematic confounds due to improper control conditions (Adachi and Willoughby, 2011; Elson et al., in press). Studies of visuospatial cognition acknowledge that action games differ from control games on multiple levels such as cognitive load, pace of action, visual demand, and motor load (e.g., Green et al., 2012). Given that most studies of visuospatial cognition employ action games with violence and control games without, violent content is another differing variable. If scholars wish to identify which variables specifically cause gains in visuospatial cognition, a systematic evaluation of games that are matched more closely on relevant variables would be necessary.

### Unclear definitions

The aggression literature uses the terminology “violent video game” whereas the visuospatial literature prefers “action game” despite studies in both realms mainly employ first-person shooter games. The terms “violent video game” and “action game” remains vague. Overall “action game” is probably preferable for both fields in avoiding unscientific emotional priming. But neither field has clarified which video games are included in such a category. Related to “violent video games,” one scholar recently commented during a murder trial that even games such as *Pac Man* could be considered violent video games (Rushton, 2013). Most would consider this absurd, and this is a serious problem of unclear delineations that potentially invite satire. The concept of action game carries less emotional load but remains unclear. Are action games only first-person shooters (the games typically used in experimental studies) or do racing or other high-paced games count?

Dye et al. (2009) make an admirable attempt at defining action games as requiring “rapid processing of sensory information and prompt action, forcing players to make decisions and execute responses at a far greater pace than is typical in everyday life” (p. 321). Yet such a definition could apply equally well to *Frogger* as it does *Call of Duty.* One of the problems with the concept of “violent video games” is that, according the vague definitions in use, *almost all video games are violent video games*. The concept of action video game would do well to avoid this trap.

## A major difference

Research on new media can often be hampered by the presence of bias among groups of scholars. Scholars who are enamored with the potential of new media may experience *savior bias*. Those who are worried about the potential negative impact of new media may experience *crusade bias* (and see Nature, 2003 for relevant comments). These processes can lead scholars, acting in good faith, to overestimate the strength, consistency, and generalizability of effects.

However, one crucial difference is the presence of societal moral panic and political pressure on the aggression agenda that is not present for visuospatial research. For instance, soon after the tragic 2012 Sandy Hook shooting, debate on video game violence which had subsided following the US Supreme Court Brown v EMA (2011) trial (in which the majority decision was highly critical of video game violence research) resumed with furor. Rep. Frank Wolf, a long-term media critic who also chairs the committee overseeing the funding of the NSF, commissioned a report on media violence and youth violence. The resultant report (Subcommittee on Youth Violence, 2013) worked hard to link media violence to mass shootings despite much evidence to the contrary, by not citing evidence conflicting with the authors' personal views. The only exception was Joanne Savage's work, miscited as supporting links between media violence and violent crime, despite that she concluded the exact opposite (Savage and Yancey, 2008). Whether the fault for this study lies with political pressure of Rep. Wolf, or *crusade bias* (and certainly citation bias) of the report authors, such advocacy-toned reports only damage the credibility of our field.

Similarly, policy statements by the American Psychological Association (2005) and American Academy of Pediatrics (2009) have been criticized for significant distortion and misstatements about the available data, typically in the direction of vastly overstating effects (Ferguson, 2013). Such professional advocacy organizations have produced policy statements by allowing scholars heavily invested in the “harm” position to review their own research and declare it beyond further debate. Given controversies over past policy statements and new research, the APA has agreed to revisit its media policy statements, which is a welcomed move. However, the committee assigned to do so consist of a majority of scholars who had taken public anti-media positions in the past. Of a total of seven, two task force members signed an amicus brief supporting the regulation of violent video games in Brown v EMA (2011), and two others have both worked closely with scholars who had helped write the previous policy statements under contention and made anti-media statements in news interviews in the past (including one who coauthored the NSF report discussed above). This tells us something crucial about policy statements: they often inform us more about the committees that write them than they do about science. Although I don't know the thinking and motives of the APA, the failure of the APA to ensure a neutral review *despite specifically being asked to do so* involves a fundamental failure of the APA policy review process, perhaps due to being overly sensitive to social moral panics and political pressure. As a consequence, a consortium of approximately 230 scholars wrote to the APA asking them to refrain from further policy statements on media and to retire their old and misleading policy statements (Consortium of Media Scholars, 2013). Psychological science must become more informed about how societal moral panics have influenced statements by scholars ranging from the 1950s comic books scare, through participation in the 1980s “Tipper Gore” hearings, to the faulty policy statements of more recent decades.

I wish to remain as positive as I possibly can and infer that these errors are the result of good faith confusion of an advocacy agenda for science. However, it becomes difficult not to see deliberate misinformation in some of these efforts. Citation bias can be a good faith result of familiarity with only certain work, or confirmation bias to which all people are prone (and I claim no exception). However, persistence in citation bias despite a history of the field being warned that it is a problem becomes more difficult to excuse as good faith. Whatever the limitations of the visuospatial cognition research may be, I see no evidence that scholars in this field have confused, purposefully, or accidentally, a cultural agenda with science, nor have I found evidence of misleading claims by scholars in this area. This may be a single difference but a critical one; one that is the distinction between science and pseudo-science.

## One rather foolish question

Sometimes I hear the question “If action games increase cognition, why can't they also increase antisocial behavior?” This question has common sense appeal, particularly for people in the general populace. But I refer to it as a “foolish question” because it is a question scientists should know better than to ask. That is because the question is a rather obvious logical fallacy, particularly when used to affirm a premise in that it relies on *false equivalence.* The logic of this question is:

**If A then B; A; hence C.**

The essence of this question (it is in fact an example of begging the question as the premise of the conclusion is critical to the question itself) is the assumption that B and C (visuospatial cognition and antisocial behavior) are equivalent. If they are equivalent, then action games effect on one should be similar as to the other. However, there is little reason to suspect that the processes that drive visuospatial cognition are equivalent to those for anti-social behavior and many reasons to suspect otherwise. Visuospatial cognition involves a straightforward cognitive practice effect, requiring no fundamental changes in personality or motivation. By contrasts advocates for action game influences on antisocial behavior have specifically posited exactly those fundamental changes to personality or motivation. For instance, Anderson and Dill (2000) suggest “If repeated exposure to violent video games does indeed lead to the creation and heightened accessibility of a variety of aggressive knowledge structures, *thus effectively altering the person's basic personality structure*, the consequent changes in everyday social interactions may also lead to consistent increases in aggressive affect” (p. 788, Italics added for emphasis). Given that the theoretical mechanisms for these two processes differ, there is no reason to assume equivalence.

Further on a more basic level, media effects are not “one size fits all” (a similar question based in false equivalence is the comparison of fictional media effects to advertising). Each individual hypothesized effect must be studied independently. Assumptions that seeing one effect must mean that all effects are true are likely to lead to gross errors and distortions within the field. In the end visuospatial cognition effects may or may not be true, and aggression effects may or may not be true, but these two sets of hypotheses must be tested independently.

## Concluding remarks

Studies of video game effects over the past few decades have labored under the cloud of social narratives regarding video games' place in society. Outcomes related to video game influences on visuospatial cognition (Boot et al., 2011) and aggression (Adachi and Willoughby, 2012) have received criticism for their methodological limitations and, perhaps, tendency to overspeak the data. Such criticisms are likely to be disappointing for researchers in the field, but they can also serve for impetus for better studies. Criticism and skepticism is an essential part of the scientific process. Fields that embrace this as part of the natural scientific process will survive. Those that do not will collapse under the weight of their own ideology.
